# TrkB/BDNF signalling patterns the sympathetic nervous system

**DOI:** 10.1038/ncomms9281

**Published:** 2015-09-25

**Authors:** Jennifer C. Kasemeier-Kulesa, Jason A. Morrison, Frances Lefcort, Paul M. Kulesa

**Affiliations:** 1 Stowers Institute for Medical Research, 1000 East 50th Street, Kansas City, Missouri 64110, USA; 2 Department of Cell Biology and Neuroscience, Montana State University, Bozeman, Montana 59717, USA; 3 Department of Anatomy and Cell Biology, University of Kansas School of Medicine, Kansas City, Missouri 64157, USA

## Abstract

The sympathetic nervous system is essential for maintaining mammalian homeostasis. How this intricately connected network, composed of preganglionic neurons that reside in the spinal cord and post-ganglionic neurons that comprise a chain of vertebral sympathetic ganglia, arises developmentally is incompletely understood. This problem is especially complex given the vertebral chain of sympathetic ganglia derive secondarily from the dorsal migration of ‘primary’ sympathetic ganglia that are initially located several hundred microns ventrally from their future pre-synaptic partners. Here we report that the dorsal migration of discrete ganglia is not a simple migration of individual cells but a much more carefully choreographed process that is mediated by extensive interactions of pre-and post-ganglionic neurons. Dorsal migration does not occur in the absence of contact with preganglionic axons, and this is mediated by BDNF/TrkB signalling. Thus BDNF released by preganglionic axons acts chemotactically on TrkB-positive sympathetic neurons, to pattern the developing peripheral nervous system.

## Abstract (web summary)

The signals that pattern the sympathetic nervous system are not fully understood. Here the authors show that the dorsal migration of the primary sympathetic ganglia in chick embryos is orchestrated by BDNF/TrkB signalling and requires contact with preganglionic axons.

## Graphical abstract

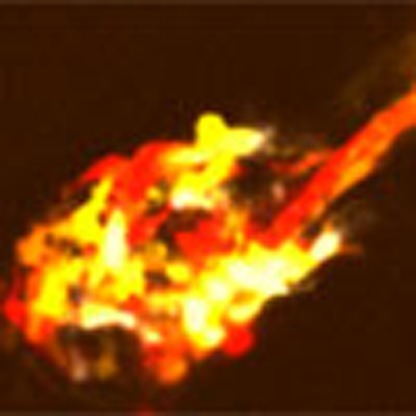

The molecular and cellular interactions that orchestrate the patterning of the vertebrate peripheral nervous system (PNS) have yet to be fully elucidated. On delamination from the neural tube, trunk neural crest cells destined to form the vertebral chain of sympathetic ganglia migrate ventrally to the vicinity of the dorsal aorta where they aggregate and condense to form the primary chain of sympathetic ganglia composed of differentiated sympathetic neurons and precursor cells^1^. Strikingly, after a pause of 24 h, these ganglia regain a mesenchymal morphology, reverse direction and migrate back dorsally in the direction from whence they originated^2^. Prevailing models of PNS development describe individual and groups of neural crest cells under the control of inhibitory signals and preprogrammed cell trajectories^3^,^4^,^5^,^6^,^7^. In such pre-pattern models, the axial level of neural tube exit and restriction to a particular migratory pathway dictates the position where neural crest cells stop and aggregate. Subsequent tissue growth moves cell clusters to a final position. The result is a reiterative pattern of discrete ganglia spaced within and ventral to the rostral somite along both sides of the spinal cord, forming the dorsal root and sympathetic ganglia, respectively. Alternative models suggest that multipotent neural crest cells respond dynamically to the microenvironmental signals that are regulated in space and time to direct emerging neural crest cells towards the dorsal aorta, sculpt individual cells into discrete clusters of primary (transient) sympathetic ganglia or halt their migration more dorsally to form dorsal root ganglia^8^,^9^,^10^,^11^. Unknown molecular signals then direct the dorsal migration of multicellular primary sympathetic ganglia to a secondary (permanent), final location^2^.

The more recent advances in molecular data on signals^12^,^13^,^14^,^15^,^16^,^17^ that appear to march along with the repeating pattern of discrete ganglia or localize near the incipient primary sympathetic ganglia before the arrival of ventral moving neural crest cells has re-awakened interest in identifying the cellular mechanisms that mediate the multi-step pattern formation of the developing nervous system. There is now a critical need for spatio-temporal information about individual and multicellular neural crest cell dynamics during nervous system development and a means to identify and accurately link gene function with local morphological changes in the tissue.

To address this challenge, we have designed novel interrogation techniques to visualize and quantify cellular and molecular dynamics during sympathetic nervous system development in living chick embryo explants. In previous work we have shown that the initial ventral migration of neural crest cells destined to form the chain of primary sympathetic ganglia do so via SDF-1/CXCR4 signalling^14^. Here our goal was to understand the multicellular migration dynamics that mediate the primary to permanent sympathetic ganglia formation and probe the role of the preganglionic sympathetic axons that extend from neuronal cell bodies in the spinal cord, during this dorsal migration.

## Results

### Coordinated dorsal migration of primary sympathetic ganglia

**Figure 1:**
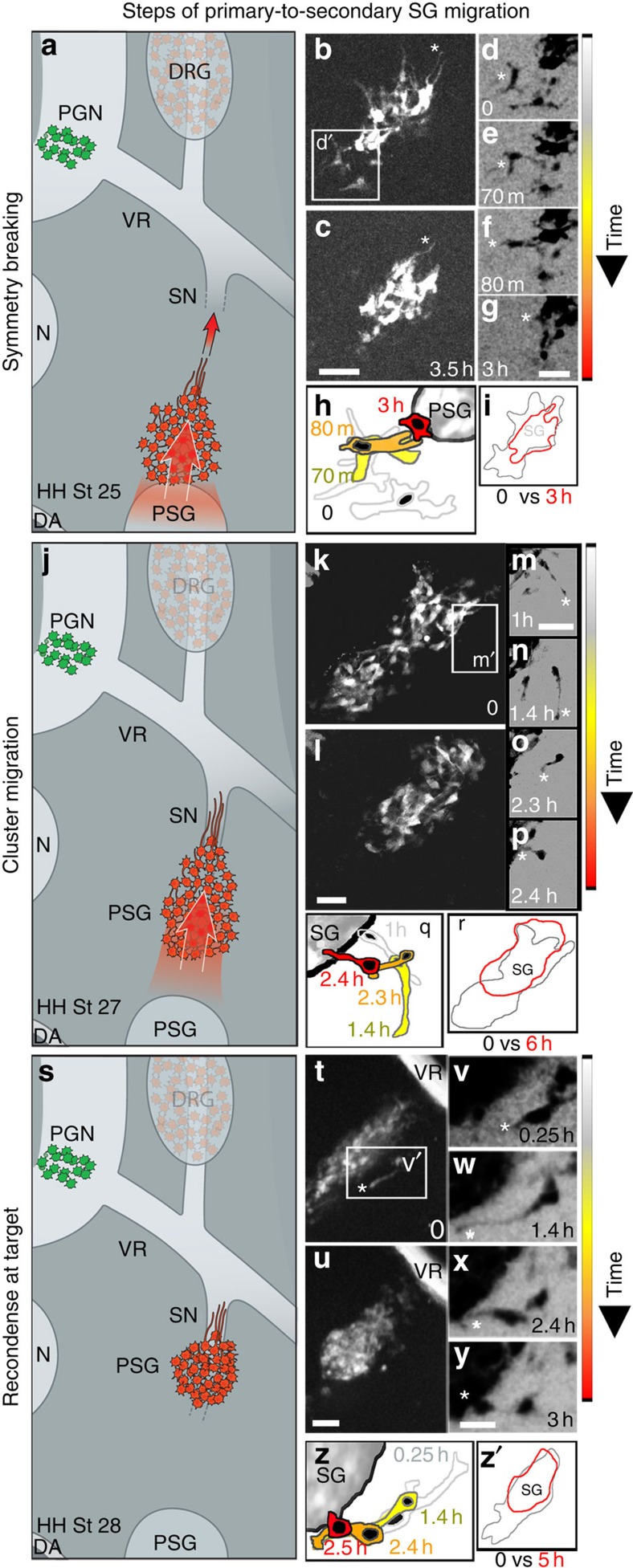
Steps of the dorsal migration of primary sympathetic ganglia. (**a**) Schematic of embryonic trunk (transverse view) from HH St. 25 chick showing the primary sympathetic ganglia (PSG, red) just after initiation to dorsal migration (termed the ‘onset of migration’) towards the ventral root (VR). Preganglionic neurons (PGNs, green) are positioned in the ventral neural tube. (**b**,**c**) Time-lapse images show that PSG break symmetry on the side facing the ventral root by extending long protrusions (**b**,**c**; asterisks) in the direction of the ventral root (top right). (**d**–**g**,**d′**) PSG cells on the side facing the dorsal aorta continue to extend short protrusions in all directions (0–3 h, asterisk). Overlay diagram (**h**) shows the positions of individual PSG cells moving to integrate into the discrete PSG cluster (grey to red) and (**i**) shape change of the PSG from 0 versus 3 h. (**j**) Schematic of a typical embryonic trunk (transverse view) from a HH St. 27 chick showing the ‘cluster migration’ step of the primary sympathetic ganglia (red) mid-route during dorsal migration. (**k**,**l**) The primary sympathetic ganglia is observed to elongate in shape, but remain cohesive. (**m**–**p**,**m′**) Individual cells that wander from the cluster find their way back to the cluster (0–2.4 h). (**q**,**r**) Overlay diagram shows an example of the wandering by an individual cell (**m**–**p**, asterisk), (**p**) return to the cluster and (**r**) shape change of the PSG over time 0 versus 6 h. (**s**) Schematic of a typical embryonic trunk (transverse view) from HH St. 28 chick showing the ‘recondense at target’ step during positioning of the final, secondary sympathetic ganglia. (**t**,**u**) The primary sympathetic ganglia is observed to become more cohesive (0–5 h) with (**v′**, asterisk; **v**–**y**) individual cells near the perimeter of the cluster re-orienting their protrusions (asterisk) and moving back into the cluster (0–3 h). (**z**,**z′**) Overlay diagrams show (**z′**) an example of the re-condensing (18% decrease in area) of the primary sympathetic ganglia (0 versus 5 h) and (**z**) movements of an individual cell to return and integrate into the cluster (0.25–2.5 h). DA, dorsal aorta; N, notochord; SN, splanchnic nerve. *n*=5 time-lapses per condition. Scale bars, 50 μm.

Time-lapse confocal microscopy of fluorescently labelled primary sympathetic ganglia cells revealed a series of well-ordered steps that appear to be choreographed to position the secondary sympathetic ganglia adjacent to the ventral root. In the first step, a small number of cells on the dorsal side of the primary sympathetic ganglia extend multiple processes restricted in orientation towards the ventral root (93% in the direction of the ventral root, calculated ±45° from the perpendicular to the ventral root; Fig. 1a–i; asterisk in b,c; Supplementary Movie 1). In contrast, cells within the ganglion that are positioned closer to the dorsal aorta continue to extend processes in all directions (56% initially in the direction of the ventral root as measured above; Fig. 1d′ (inset); d,e, asterisk; Supplementary Movie 1); although they subsequently reorient the majority of their processes in the direction of the ventral root (Fig. 1f,g, asterisk; Supplementary Movie 1) and move into the body of the loosely connected cluster (Fig. 1c,h,i; Supplementary Movie 1). We identify this step as symmetry breaking of the primary sympathetic ganglia.

In the subsequent step, primary sympathetic ganglia elongate and move as a coordinated group (Fig. 1j–r, Supplementary Movie 2). Individual cells within the loosely connected cluster maintain extensive contacts with both local and non-local (more than one cell diameter) neighbours (Fig. 1k,l; Supplementary Movie 2). Cells that move away from the cluster (Fig. 1m′(inset), m–q, Supplementary Movie 2) may extend long processes to sample the local microenvironment (Fig. 1m,n, asterisk; q, Supplementary Movie 2), but markedly collapse, reorient processes (Fig. 1o, asterisk; q, Supplementary Movie 2), and move to re-join the loosely connected cluster (Fig. 1p, asterisk; q, Supplementary Movie 2). In addition, a fiduciary mark placed in the tissue (Supplementary Movie 2; upper left purple marking) confirms sympathetic ganglia cell clusters migrate past this stationary point in the tissue, indicating active migration and not solely tissue growth results in positioning the permanent sympathetic ganglia. We identify this step as ‘directed migration of the primary sympathetic ganglia’.

The last step completes the positioning of the primary sympathetic ganglia cluster, thus conferring ‘secondary’ status to the ‘primary’ sympathetic ganglia. As the primary sympathetic cluster approaches the ventral root, cells change shape and position to reform a tightly packed, cohesive ganglion (Fig. 1s,z′, 18% decrease in size; Supplementary Movie 3). Individual cells near the perimeter of the cluster (Fig. 1t,v′(inset), v; asterisk, Supplementary Movie 3) reorient their processes (Fig. 1w–y, asterisk; z, Supplementary Movie 3) and move to integrate into the cluster (Fig. 1y, asterisk; z, Supplementary Movie 3). This is the ‘re-aggregation of the primary sympathetic ganglia at target’. Thus, the migration event of the primary sympathetic ganglia to the secondary, final location is a well-coordinated, multi-step process that includes symmetry breaking, directed migration of a loosely connected and coordinated cell group and re-aggregation into a tight cell cluster.

### Post- and preganglionic neurons extensively interact

**Figure 2:**
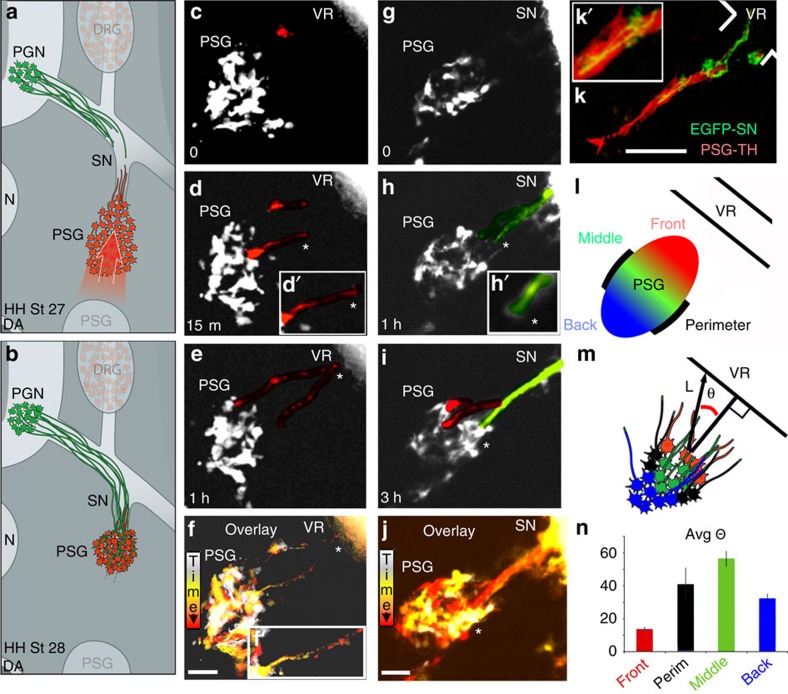
Sympathetic ganglia require contact with preganglionic neurons. (**a**,**b**) Schematic representations (transverse view) from HH St. 27–28 embryos showing dorsally migrating primary sympathetic ganglia (PSG; red) and preganglionic sympathetic neurons (PGN cell bodies within the ventral neural tube) extending down the ventral root (VR) and branching off in the splanchnic nerve (SN). (**c**–**e**) Images from a typical time-lapse session showing cell protrusions from PSG (**d**,**d′**; asterisk) towards the VR and (**f**) overlay of the time-lapse images, colour coded by time with higher magnificaiton of extension shown in f’ (grey=0; red=1 h). (**g**–**i**) Images from a typical time-lapse imaging session showing extensions from preganglionic sympathetic neurons (asterisk) in the VR turning through the SN to interact with dorsally migrating PSG cells (**h**,**h′**,**i**; asterisk). (**j**) Overlay of the time-lapse sequence, colour coded by time (grey=0; red=3 h). (**k**) Projected image from a three-dimensional confocal z-stack showing the physical interaction between preganglionic axons (green, ventral neural tube electroporation) and dorsally migrating PSG cells (red, dorsal neural tube electroporation) at HH St. 26 with higher magnification of interactions shown in k’. (**l**–**n**) Measurements of PSG protrusions. The angle of a cellular protrusion was measured from the line drawn from the approximate centre of the PSG to the perpendicularly bisect the VR. Cells were categorized by position within the PSG; front (red), perimeter (black), back (blue) and middle (green). DA, dorsal aorta; DRG, dorsal root ganglia; N, notochord; PGN, preganglionic neuron. *n*=5 time-lapses per condition. Scale bars, 50 μm.

Given the selective orientation of processes extending from the primary sympathetic ganglia towards the developing spinal nerve immediately before dorsal migration, we sought to investigate the dynamic interactions between the dorsal migrating post-ganglionic cells with the spinal nerve. The first signs of cellular interaction between the pre- and post-ganglionic neurons occur on the dorsal, or spinal nerve facing, side of the primary sympathetic ganglia (Fig. 2a–k, Supplementary Movie 4). Cells within the primary sympathetic ganglia extend long protrusions that stretch over 100 μm and persist in the direction of the spinal nerve (Fig. 2d–f, d′; asterisk; Supplementary Movie 4). Long protrusions extend from all cells within the primary sympathetic ganglia, but preferentially from neural cells within the middle of the cluster (Fig. 2l–n, Supplementary Movie 4). In comparison, preganglionic sympathetic axons that course through the ventral root make a stunning 90° turn towards the primary sympathetic ganglia (Fig. 2b; splanchnic nerve, Supplementary Movie 5), before physical contact with primary sympathetic ganglia cells (Fig. 2b,g,h,h′; asterisk, Supplementary Movie 5). Growth cone tips of the preganglionic sympathetic neurons continue to extend and glide along the primary sympathetic ganglia neuronal protrusions (Fig. 2i, Supplementary Movie 5), eventually stopping when they reach the primary sympathetic ganglia cell bodies (Fig. 2b,i,j; asterisk, Supplementary Movie 5). Although the primary sympathetic ganglia structure is loosely cohesive, the cells within are still moving dynamically while in contact with preganglionic axonal projections. In time-lapse Supplementary Movies, preganglionic axonal projections intertwine between migrating primary sympathetic ganglia neurons (Fig. 2k, Supplementary Movie 5) to eventually circumscribe the multicellular cluster and move with it to the final, permanent location adjacent to their branch point off the ventral root (Supplementary Movie 5).

### Dorsal migration requires contact with preganglionic axons

**Figure 3:**
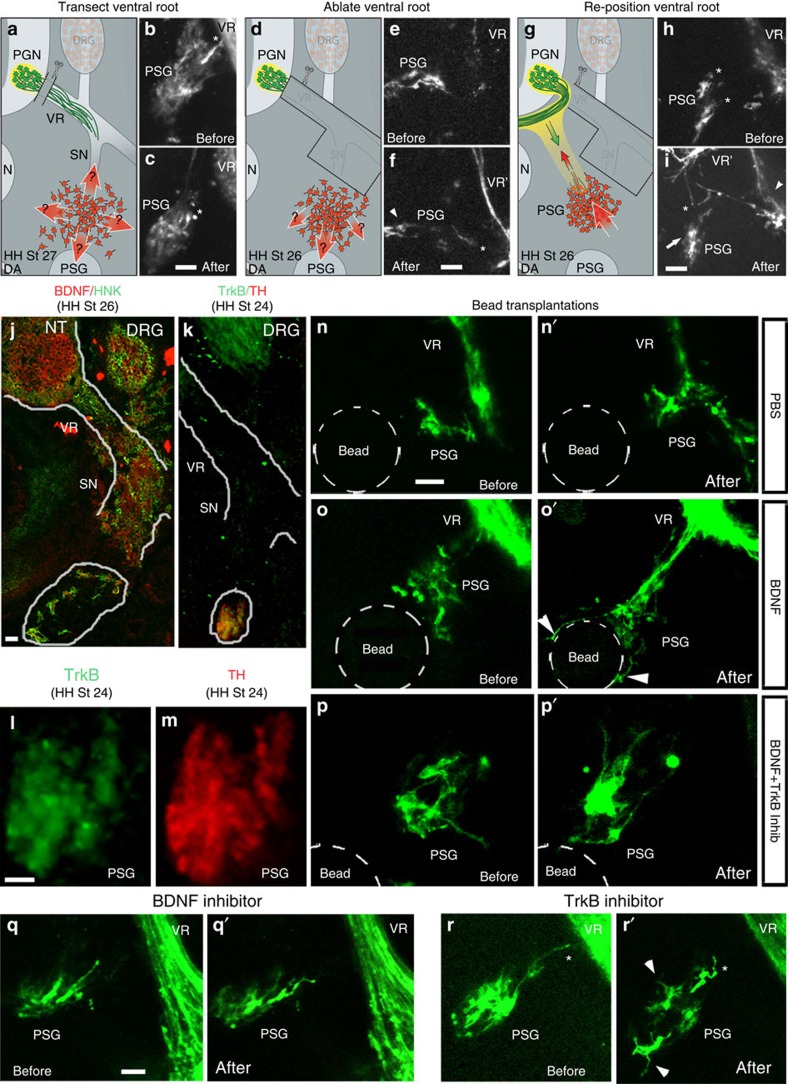
Manipulation of ventral root or TrkB signalling disrupts migration. (**a**) Schematic of ventral root transection (HH St. 27) and two images from a typical time-lapse imaging session showing (**b**, asterisk) primary sympathetic ganglia contact with the ventral root and (**c**, asterisk) retraction of primary sympathetic ganglia protrusions. (**d**) Schematic of ventral root removal (HH St. 26) and images from a typical time-lapse imaging session showing (**e**) the collapse of the primary sympathetic ganglia and (**f**) re-extension of cell protrusions towards the regrowth of the ventral root (asterisk) and dorsal aorta (arrowhead). (**g**) Schematic of the re-positioning of the ventral root and two images from a typical time-lapse imaging session showing (**h**) the retraction of primary sympathetic ganglia protrusions (asterisks), from the cell bodies (arrow), (**i**) re-extension of protrusions in the direction of the ectopic ventral root (asterisk) and regrowth along the ventral root (arrowhead). (**j**) BDNF (red) protein is expressed in the ventral neural tube and ventral root, shown here in a typical HH St. 24 transverse trunk section with HNK (green). (**k**) TrkB (green) protein is expressed in primary sympathetic ganglia in a typical HH St. 24 transverse trunk section with tyrosine hydroxylase (TH; red). (**l**,**m**) Higher magnification of primary sympathetic ganglia in (**k**) showing trkB (green) and TH (red) in separate panels. (**n**,**n′**) PBS-control-soaked bead (HH St. 25) has no effect on dorsal migrating sympathetic ganglia. (**o**,**o′**) BDNF-soaked beads (HH St. 25) re-direct dorsal migrating primary sympathetic neurons. A BDNF-soaked bead placed distal to dorsal migrating sympathetic ganglia attracts cellular extensions and maintains interactions with sympathetic neurons. (**p**,**p′**) BDNF-soaked bead (HH St. 25) in the presence of TrkB inhibitor has little affect on primary sympathetic ganglia. (**q**,**q′**) Before and after images of the primary sympathetic ganglia in the presence of a BDNF inhibitor (HH St. 25; 5 μg ml^−1^ of blocking BDNF antibody) and (**r**,**r′**) TrkB function blocking antibody (HH St. 25) with retraction of the primary sympathetic ganglia cell protrusions (**q**,**q′**,**r**,**r′**; asterisk). DA, dorsal aorta; DRG, dorsal root ganglia; N, notochord; PGN, preganglionic neuron; PSG, primary sympathetic ganglia; SN, splanchnic nerve; VR, ventral root. *n*=5 time-lapses per condition. Scale bars, 50 μm.

Given the tight and persistent contact between the dorsally migrating primary sympathetic ganglia and preganglionic axons, we sought to determine whether this contact was required for dorsal migration. To this end, we manipulated the ventral root and tracked the cell dynamics of the primary sympathetic ganglia (Fig. 3a–i). Transection (Fig. 3a–c, Supplementary Movie 6) or removal (Fig. 3d–f, Supplementary Movie 7) of the ventral root from the base of the ventral neural tube after the primary sympathetic ganglia initiated dorsal migration Hamburger and Hamilton (HH) St. 25; 40, caused the primary sympathetic ganglia to stall (compare Fig. 3b with Fig. 3c, lack of movement to final destination) and fail to reach its final position (Fig. 3c,f, Supplementary Movies 6 and 7). Although the primary sympathetic ganglia stalled mid-route, their initial direction of migration was not altered (Fig. 3c,f, Supplementary Movies 6 and 7). Cell protrusions were shorter and not limited to orientation towards the ventral root (Fig. 3c, asterisk; f, asterisk, Supplementary Movies 6 and 7). Rather than moving dorsally, primary sympathetic ganglia cells on the trailing side of the multicellular cluster extended projections and moved in the opposite ventral direction towards the dorsal aorta (Fig. 3f, arrowhead, Supplementary Movies 6 and 7). In some cases, cells within the stalled primary sympathetic ganglia re-directed long cellular protrusions towards new axonal projections emerging from the regenerating ventral root (Fig. 3f, asterisk, Supplementary Movie 7). This resembled a tug-of-war between the leading and trailing edges of the elongated multicellular primary sympathetic ganglia cluster, causing it to disassemble into individual moving cells (Fig. 3f; arrowhead versus asterisk, Supplementary Movie 7).

When the ventral root was cut and repositioned to an ectopic location within the (inhibitory) perinotochordal area, the behaviour of primary sympathetic ganglia cells was dramatically altered (Fig. 3g–i, Supplementary Movie 8). Initially the primary sympathetic ganglia cluster stalled along the migratory pathway (Fig. 3h, Supplementary Movie 8). Cells within the cluster displayed short, rapid protrusions in multiple directions (Fig. 3h, asterisks, Supplementary Movie 8). A few leading edge cells on the side towards the original ventral root reached out to the distal portion of the ventral root where some tissue is still intact. However, the majority of cells moved away from the cluster with circuitous trajectories in the direction towards the repositioned ventral root or to its original location (Fig. 3i, Supplementary Movie 8). Individual primary sympathetic ganglia cells that migrated to within range of the repositioned ventral root, markedly responded by extending long protrusions, in the direction of the new ventral root location (Fig. 3i, VR′, Supplementary Movie 8) to establish stable contacts (Fig. 3i, asterisk, Supplementary Movie 8). Axonal projections from the repositioned ventral root extended towards the incoming individual primary sympathetic ganglia cells (Fig. 3i, asterisk, Supplementary Movie 8) and along the original ventral root (Fig. 3i, arrowhead, Supplementary Movie 8). Cells on the ventral side of the primary sympathetic ganglia extended protrusions back towards the dorsal aorta, in the process stretching out the ganglia cluster in axis between the repositioned ventral root and dorsal aorta (Fig. 3i, arrow, Supplementary Movie 8). Thus, there is a dynamic interplay between the primary sympathetic ganglia and preganglionic axons involving a mutual attraction that is required to position the permanent, secondary sympathetic ganglia. Since in response to ventral root transections, Schwann cells are present but sympathetic ganglia dorsal migration is impaired, this argues against the possibility that Schwann cells resident within the ventral root are responsible for secreting a sympathetic ganglia attractant. Instead, these data argue for a strong attractive signal associated with preganglionic axons.

### Molecular control of sympathetic ganglia positioning

To investigate the molecular cues mediating the tight relationship between the primary sympathetic ganglia and ventral root, we used laser capture microdissection coupled with quantitative polymerase chain reaction (qPCR) gene profiling^18^,^19^. This analysis revealed a panel of unique chemoattractant signals present in the ventral root during the successive stages of primary sympathetic ganglia dorsal migration. Comparison of receptor gene expression (qPCR) by primary sympathetic ganglia cells showed a temporal correlation between brain-derived neurotrophic factor (BDNF), expressed by the ventral neural tube at HH St. 24 (4.2 times higher in the ventral neural tube versus sympathetic ganglia immediately before the onset of dorsal migration; Supplementary Fig. 1d), and TrkB, expressed by cells within the primary sympathetic ganglia at HH St. 24, before the initiation of dorsal migration (Fig. 3l,m; TrkB is expressed 2.3 times higher in sympathetic ganglia versus ventral neural tube using qPCR, Supplementary Fig. 1c). This was further confirmed by protein localization (Fig. 3j–m). TrkB protein was shown to be strongly expressed by HH St. 24 sympathetic ganglia but absent in the ventral root (Fig. 3k–m). BDNF was strongly expressed in the ventral neural tube at HH St. 24 (immediately before the onset of dorsal migration, location of preganglionic neurons; Fig. 3j) and ventral root. BDNF protein expression did not overlap with Schwann cells in the ventral root, but this does not rule out the possibility that they also produce BDNF.

To further define the cell populations expressing BDNF, we performed *in situ* hybridization on E4 trunk tissue for BDNF mRNA and found expression in dorsal root ganglia, medial neural tube, ventral neural tube and ventral root (Supplementary Fig. 1e,f,h). Antibody staining for the Schwann cell marker Cad7 showed slight overlap with BDNF mRNA at the ventral root exit zone (Supplementary Fig. 1g). Similarly, antibody staining for the neural marker Tuj1 showed overlap with BDNF mRNA in the dorsal root ganglia, ventral neural tube (location of preganglionic neurons) and ventral root (Supplementary Fig. 1i), indicating both Schwann cells and axons in the ventral root express BDNF. Since Schwann cells and axons cells within the ventral root sit in close juxtaposition and BDNF whole embryo *in situ* hybridization did not give single-cell resolution, we did fluorescence-activated cell sorting analysis on P0 (Schwann cell marker)-sorted cells and ventral neural tube-dissected tissue and performed qPCR for BDNF mRNA. We found BDNF mRNA expression in the ventral neural tube (as expected) and in the Schwann cell population in the ventral root (Supplementary Fig. 1j). Together, these data indicate multiple sources of BDNF signalling to attract dorsally migrating sympathetic ganglia. Support for these data in the chick include *in situ* hybridization studies for BDNF that confirm mRNA expression in the ventral neural tube^20^. In addition, Wetmore and Olson^21^ show BDNF protein expression in sympathetic ganglia although BDNF mRNA is absent. In contrast, primary sympathetic neurons have been shown to express TrkB mRNA^21^,^22^.

To determine the functional significance of TrkB/BDNF signalling during formation of the secondary chain of sympathetic ganglia, primary sympathetic ganglia cells were challenged with an ectopic source of BDNF during dorsal migration (Fig. 3n–p’, Supplementary Movie 9). Positioning of BDNF-coated beads in a location in which the primary sympathetic ganglia normally avoid, induced rapid protrusive growth in that direction (compare Fig. 3n′ with o′, Supplementary Movie 9). This was followed by cell body translocation towards the ectopic BDNF source (Fig. 3o′, arrowheads, Supplementary Movie 9), which was not observed in response to PBS-coated beads (Fig. 3n′). These behaviours were reminiscent of the dynamics observed when primary sympathetic ganglia cells moved through the perinotochordal region to locate the repositioned ventral root (Fig. 3g–i, Supplementary Movie 8). This response could be abrogated by the addition of a Trk-B-function-blocking antibody, which inhibits TrkB signalling (Fig. 3p,p′), in which case, primary sympathetic ganglia cells failed to respond to either the ectopic BDNF source and/or endogenous BDNF from the splanchnic nerve and ceased directed migration towards the splanchnic nerve (Fig. 3r,r′). Further support for a critical role for TrkB/BDNF signalling was the cessation of primary sympathetic ganglia dorsal migration in the presence of a blocking BDNF antibody, (Fig. 3q,q′, Supplementary Movie 10). Furthermore, solely in the presence of the TrkB inhibitor, primary sympathetic ganglia neurons retracted their processes directed at the ventral root (Fig. 3r,r′; asterisk), extended shorter dynamic processes in all directions (Fig. 3r′, arrowheads) and failed to condense and complete dorsal migration (Fig. 3r,r′).

**Figure 4:**
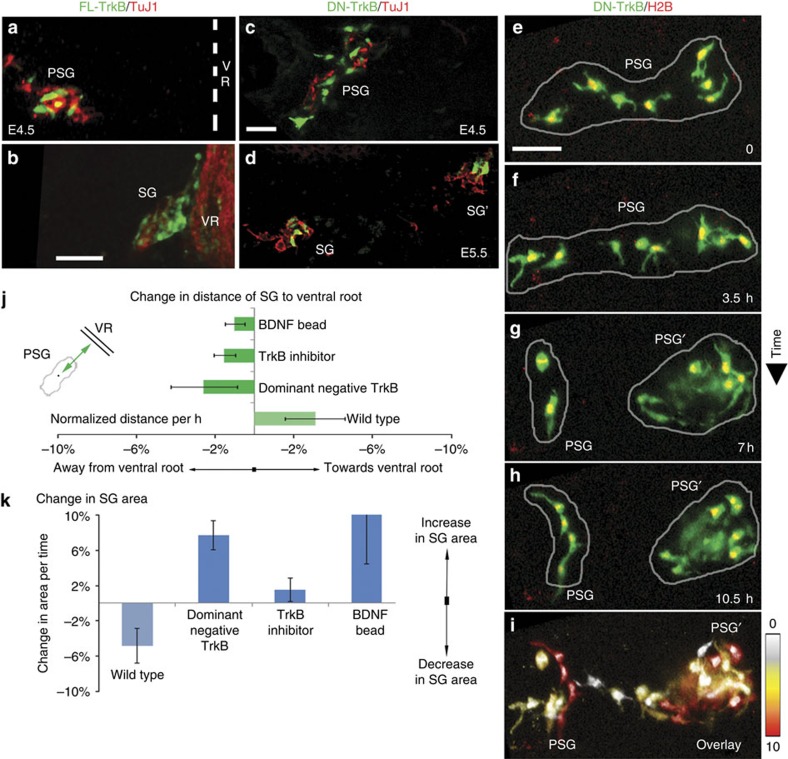
Altering trkB signalling disrupts dorsal migration. (**a**,**b**) Over-expression of FL-TrkB-EGFP results in properly formed PSG at E4.5 and secondary permanent ganglia at E5.5. (**c**,**d**) Knockdown of TrkB using DN-TrkB-EGFP results in proper PSG localization and initiation of dorsal migration (E4.5) but failure to form proper permanent ganglia (E5.5) with some cells remaining at the primary site (**d**, labelled SG) and others distributed along the dorsal migratory path or reaching their permanent location (**d**, labelled SG′). (**e**–**i**) Time-lapse analysis of DN-TrkB-EGFP-expressing sympathetic ganglia cells during dorsal migration. Cells are shown to initiate migration from the primary site, but shortly after the onset of migration the cluster breaks apart with some cells continuing towards the secondary site while other cells reverse direction and return to the primary site. (**i**) Time points from a typical time-lapse imaging session (**e**–**h**) are colour coded and overlaid. (**j**) Graph of the change in distance of PSG to the ventral root wild-type PSG (light green bar) move towards the ventral root, while in the presence of trkB inhibitor, dominant negative trkB or BDNF bead the PSG clusters move away from the target site (dark green bars). (**k**) Graph of change in PSG area. Wild-type PSG (light blue) condense and decrease their ganglia size, whereas in the presence of trkB inhibitor, dominant negative trkB or BDNF bead fail to condense, and increase their ganglia size (dark blue bars). DA, dorsal aorta; DRG, dorsal root ganglia; N, notochord; PGN, preganglionic neuron; PSG, primary sympathetic ganglia; SN, splanchnic nerve; VR, ventral root. *n*=5 time-lapses or embryos per condition. Scale bars 50 μm.

To further define the role of TrkB/BDNF signalling during primary sympathetic ganglia dorsal migration, we selectively altered TrkB expression in primary sympathetic ganglia cells by electroporating pre-migratory neural crest cells with either a full-length TrkB construct expressing enhanced green fluorescent protein (FL-TrkB-EGFP) or a dominant negative TrkB construct expressing EGFP (DN-TrkB-EGFP) (Fig. 4). Tracking of DN-TrkB transfected primary sympathetic ganglia cells by time-lapse confocal microscopy revealed that the primary sympathetic ganglia arose normally in their typical ventral location adjacent to the dorsal aorta (Fig. 4a,c). This was expected as TrkB expression was not detected until after primary sympathetic ganglia coalesced (Fig. 4a,c). Over-expression of FL-TrkB yielded normal primary, but also secondary sympathetic ganglia formation (Fig. 4a,b).

Conversely, the DN-TrkB embryos showed that dorsal migration was drastically impaired, with proper secondary sympathetic ganglia failing to form adjacent to the splanchnic nerve (Fig. 4c,d). DN-TrkB-expressing cells initiated migration from their primary location at the dorsal aorta and moved in the direction of the ventral root, but stalled shortly after their departure from the dorsal aorta (Fig. 4e–i). The multicellular primary sympathetic ganglia cluster separated into a minority population (Fig. 4e) that reached the ventral root (Fig. 4f,g), with some cells remaining at the primary site (Fig. 4g,h), and the remainder of cells that initiated migration stalling en route to the ventral root (Fig. 4h). Intriguingly, even though cells failed to reach their secondary, permanent location, primary sympathetic ganglia cells transfected with DN-TrkB still condensed and attempted to form ganglia in ectopic locations (Fig. 4i).

Quantification of the distance primary sympathetic ganglia cells migrated towards the ventral root after alterations in TrkB expression, demonstrated the dramatic extent to which cells failed to reach their permanent location (Fig. 4j). Measurements of primary sympathetic ganglia cluster shape supported the time-lapse observations that reduction of TrkB expression resulted in a less condensed cell cluster and failure of cells to re-aggregate (Fig. 4k). Together, these data reveal that directed cell migration and secondary sympathetic ganglion condensation are controlled by two unique mechanisms, as we have previously reported for primary sympathetic migration and condensation^14^,^23^,^24^.

## Discussion

The dynamic response of primary sympathetic ganglia cells to TrkB/BDNF chemotactic signals associated with preganglionic sympathetic axons, controls the permanent sympathetic ganglia pattern. This study demonstrates that primary to secondary sympathetic ganglia migration involves a transition from individual to coordinated cell migration. Our time-lapse imaging from show this occurs in a series of well-coordinated steps that include symmetry breaking, directed migration, then re-aggregation of cells within the primary sympathetic ganglia at the final permanent location adjacent to the spinal nerve (Figs 1 and 2). Our findings support and significantly enhance the static observations of Kirby and Gilmore (who first described a secondary migration of sympathetic ganglia based on static imaging from Cornbrooks *et al*.^25^), and their observation that the timing of dorsal migration of sympathetic ganglia correlated with the outgrowth of preganglionic axons from the ventral root, forming the splanchnic nerve. In fact, we show by tissue manipulation that there is a critical interplay between the primary sympathetic ganglia and preganglionic sympathetic axons to position the secondary sympathetic ganglia (Fig. 3), suggesting dynamic molecular signals underlie the final positioning of the secondary sympathetic ganglia rather than tissue growth. Intriguingly at the same time as their axons are inducing the directed migration of primary sympathetic ganglia, the cell bodies of the preganglionic sympathetic neurons are themselves migrating dorsally within the ventral spinal cord to form the mature Column of Terni^8^,^26^,^27^.

While TrkB/BDNF signalling is critical for synaptic function and plasticity, its role in chemotaxis^28^ has been shown previously in the developing cortex and cerebellum *in vitro*^29^ and *in vivo*^30^. BDNF is also required for target innervation and patterning of sensory innervation^31^,^32^,^33^. Before our study, the function of TrkB during normal sympathetic ganglia development was unclear. Previous reports of *Trk*b^−/−^ or *B*dnf^−/−^ mice failed to show a defect in sympathetic ganglia, however, these reports used superior cervical ganglia for their investigations (somite levels 5–10; not thoracic/trunk sympathetic ganglia levels at somite^18^,^19^,^20^,^21^,^22^,^23^,^24^,^34^,^35^,^36^). Furthermore, TrkB expression has previously been reported in lumbar sympathetic ganglia but not superior cervical ganglia^22^, further indicating the superior cervical ganglia is not a good predictor of trunk sympathetic ganglia behavior. Finally, BDNF has been reported to be expressed by mouse sympathetic ganglia at E14.5, however, this equates to chick day 7, well after sympathetic ganglia dorsal migration is complete^37^. Interestingly, mice in which *Bdnf* is deleted do not form normal synaptic connections between preganglionic axons and sympathetic ganglia^34^ although the mechanisms responsible for that failure have not been determined. Our data would indicate that this deficit results from a failure in BDNF-induced positioning of sympathetic ganglia.

**Figure 5:**
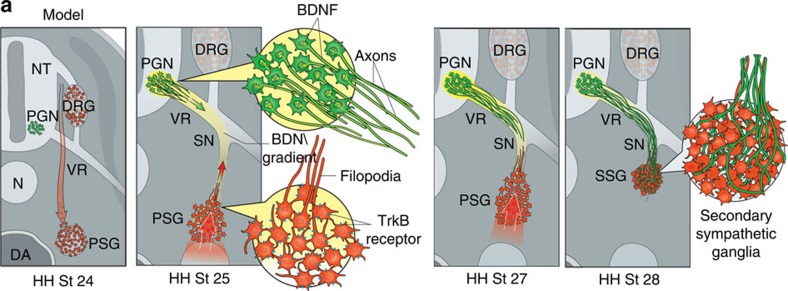
Model of the dorsal migration process of sympathetic ganglia. (**a**) Model of the cellular and molecular choreography underlying the development of the sympathetic nervous system that includes the dorsal migration process of the primary sympathetic ganglia to form the final secondary sympathetic ganglia.

Our novel findings of TrkB/BDNF signalling playing a chemoattractant role for sympathetic nervous system formation is intriguing in light of recent research on neuroblastoma, a paediatric tumour of the peripheral nervous system that arises from sympatho-adrenal lineages of the neural crest^38^. Neuroblastomas high in TrkA often spontaneously regress and show good prognosis, whereas those high in TrkB grow aggressively and are highly invasive^38^,^39^. Here we have defined a novel role for TrkB/BDNF chemoattraction signalling during a period of sympathetic nervous system development (Fig. 5), which could highlight a critical time period in development when the early transformation from normal neuroblast to neuroblastoma occurs.

## Methods

### Embryos

Fertilized White Leghorn chicken eggs (Ozark Hatchery, Meosho, MO) were placed in a incubator at 37 °C (Kuhl, Flemington, NJ) until appropriate age of development. Eggs were rinsed with 70% alcohol, 3 ml albumin was removed and windowed and staged according to Hamburger and Hamilton (HH, 1951)^40^. Embryos were injected in ovo at HH St. 10 with an EGFP-encoding plasmid: pMES full-length or DN-trkB-EGFP constructs to fluorescently label pre-migratory neural crest cells located in the dorsal neural tube, using a borosilicate glass capillary pulled needle (World Precision Instruments, MTW100-4). pMES is a control EGFP empty vector that utilizes the chick beta-actin promoter and internal IRES site. Fast Green FCF (Sigma, F-7252; 10 μg ml^−1^) was added 1:5 to the injection needle to visualize injection of the constructs. Constructs were electroporated into pre-migratory neural crest cells using gold-coated Genetrode elextrodes (Fisher, BTX512) and electroporator (Genetronics, San Diego, CA) with five 50-ms pulse of 20 V. Eggs were resealed with adhesive tape and incubated at 38 °C for 3–4 days. After incubation, we evaluated each embryo before manipulation for brightness and uniformity of EGFP label using a fluorescein isothiocyanate (FITC) filter to observe GFP-positive cells Axiovert microscope (Carl Zeiss, Thorwood, NY) with a 10 × /0.3 objective (Carl Zeiss). We selected embryos that were healthy and well labelled.

### Gene profiling

Tissue was collected by laser capture microdissection (LCM; Carl Zeiss; Supplementary Fig. 1a), pre-amplified using a miniaturized version of the Cells-to-Ct kit (Ambion, Life Technologies, Grand Island, NY) and analysed by microfluidic RT–qPCR on the BioMark HD (Fluidigm, South San Francisco, CA). Following LCM, RNA from residual cryosections produced RINs of 6.4–9.4 on a Bioanalyzer 2100 (Agilent, Santa Clara, CA). A total of 96 transcripts were pre-amplified from cDNA using 14 cycles per the Cells-to-Ct protocol (Life Technologies). Pre-amplified cDNAs were diluted with sterile 1 × TE and the products were analysed on a Fluidigm BioMark HD at the Children’s Hospital Boston IDDRC Molecular Genetics Core. No Template negative control reactions failed to produce signal. Median absolute deviation was used to eliminate outliers, resulting in at least three biological replicates per sample. A trio of reference genes, selected from eight candidates, was used to calculate normalized relative quantities. Statistical analyses were performed with RealTime StatMiner (Integromics, Waunakee, WI). Alternatively, cells of interest were sorted directly into Cells-to-Ct lysis solution (Ambion, Life Technologies) by cytometry. cDNA was synthesized directly from lysates, pre-amplified using a miniaturized version of the Cells-to-Ct kit (Ambion, Life Technologies) and analysed by RT–qPCR on an ABI7900HT (Applied Biosystems, Waltham, MA).

### *In vitro* cultures

Embryos were injected and electroporated with an EGFP plasmid (pMES) at HH St. 10 and incubated for 72 h. Embryos were then sectioned transversely, using a feather blade (∼150-μm thick), between the fore- and hindlimbs and placed on a Millicell culture insert (EMD Millipore, Billerica, MA). BDNF- (Peprotech, Rocky Hill, NJ) or PBS-soaked Affigel beads (Bio-Rad, Hercules, CA) were placed in the slices along the dorsal migratory path of neural crest cells but in ectopic locations that the cells normally avoid (that is, adjacent to notochord, ventral to spinal nerve, dorsal aorta), using fine glass pipettes and tungsten needles. The Millipore filter was then placed in a glass bottom Petri dish and tissue time-lapse imaged. For function-blocking experiments, trunk transverse tissue sections were cultured as above and the following function blocking antibodies were added to the culture media: anti-BDNF (1:500; Millipore, #AB1513P) or anti-trkB (1:500; R and D systems, #AF1494).

### Immunohistochemistry

Embryos were collected in PBS and fixed in 4% paraformaldehyde (PFA) for 4 h. Embryos for immunohistochemistry were fixed as above and processed as in Kasemeier-Kulesa *et al*.^24^. Primary antibodies included: HNK-1 (1:500, NC cell marker; American Type Culture Collection, #TIB-200), tyrosine hydroxylase (TH; 1:50; sympathetic neurons; DSHB, University of Iowa, #aTH), trkB (1:200; BioVision, #3593-100), BDNF (1:250; Aviva Systems Biology, #ARP41970-P050), Tuj1 (1:500, neural; R and D Systems, #MAB1195), P0 (1:50, Schwann cells; DSHB, University of Iowa, #1E8) and Cad7 (1:20, Schwann cells; DSHB, University of Iowa, #CCD7-1).

### Full-length and DN-trkB construction

The entire coding sequence of chick TrkB (F. Lefcort) was subcloned into the EGFP-expressing vector, pMES to generate FL-chTrkB-GFP. A dominant negative form of the receptor was generated using PCR with primers to amplify the region from the start codon, and terminating immediately after the transmembrane domain, before the phospho-tyrosine signalling domains. Forward primer 5′-GAATTCATGGTGTCCTGGCGGCGGAG-3′ and reverse primer 5′-CCCGGGTTATGTCATCCCAATGATGACCGCAT-3′. This segment was then subcloned into the EGFP-expressing vector, pMES to generate DN-TrkB-GFP.

### Probe generation

The chick BDNF *in situ* probe was generated by designing a gene fragment (gBlock, Integrated DNA Technologies) to the chick BDNF gene incorporating EcoRI restriction site at the 5′-end and BamHI at the 3′end (NCBI Accession #NM_001031616) and inserted into pDrive cloning vector at the EcoRI and BamHI sites (Qiagen). For the antisense probe, vector was linearized with Kpn1 and synthesized with SP6 RNA polymerase. For the sense probe, vector was linearized with Not1and synthesized with T7 RNA polymerase.

### *In situ* hybridization

Embryos were collected and fixed as above and dehydrated in a methanol gradient and store at −20°C. Embryo were then rehydrated and vibratome sectioned at 100 μm under RNase free condition. *In situ* hybdrization was carried out as in Wilkinson^41^. Briefly, probes were incubated overnight, Antidigoxigenin-AP fragment (Roche), 1:2,000, was applied to sections overnight and washed. Sections were developed with BM Purple (Roche) for 2–4 h for colour reaction to develop. Sections were then stained for Tuj1 (neural) and Cad7 (Schwann) markers using IHC noted above.

### Three-dimensional confocal and time-lapse imaging

Three-dimensional image z-stacks were collected on an inverted laser scanning confocal microscope (LSM5 Pascal, Carl Zeiss) using either a Plan-Neofluar 10 × /0.3, Plan-Neofluar 40 × /0.75 or C-Apochromat 40 × /1.2 W objective (Carl Zeiss). For embryo explant time-lapse microscopy, the microscope was surrounded with a snug-fitting cardboard box and thermal insulation (Reflectix, BP24025, Markelville, IN) with a table-top incubator (Lyon Electric, 950-107, Chula Vista, CA) fed into one side of the box (Kulesa and Kasemeier-Kulesa, 2007). The EGFP plasmid was excited with the 488-nm laser line using the FITC filter. Time-lapse images were recorded every 5 min for an average of 12–16 h. Images were collected, processed and analysed using AIM software (Carl Zeiss) and ImageJ v1.30 software (developed at NIH and available on the Internet at http://rsb.info.nih.gov/ij/). Statistical analysis was performed using the Student’s *t*-test.

### Image analysis

Area calculations were made by masking the region covered by GFP+ SG cells and using Imaris software (Bitplane) to calculate the area covered.

For distance of SG cluster to ventral root, the centre of mass of the area determined above was used to measure the distance to the perpendicular of the ventral root on static images from time-lapse session.

## Additional information

**How to cite this article:** Kasemeier-Kulesa, J. C. *et al*. TrkB/BDNF signalling patterns the sympathetic nervous system. *Nat. Commun.* 6:8281 doi: 10.1038/ncomms9281 (2015).

## Supplementary Material

Supplementary Information Supplementary Figure 1

Supplementary Movie 1 Symmetry Breaking of the Primary Sympathetic Ganglia Cluster

Supplementary Movie 2 Cluster Migration of the Primary Sympathetic Ganglia. Sympathetic ganglia labeled with cytoplasmic (yellow) and nuclear (purple) label. Fiduciary mark (upper left hand corner in purple).

Supplementary Movie 3 Re-aggregation of the Primary Sympathetic Ganglia at the Ventral Root

Supplementary Movie 4 Dynamic Interplay of the Primary Sympathetic Ganglia Towards the Ventral Root

Supplementary Movie 5 Dynamic Interplay of the Pre-Ganglionic Axon Projections with the Primary Sympathetic Ganglia

Supplementary Movie 6 Transection of the Ventral Root and Response of the Primary Sympathetic Ganglia

Supplementary Movie 7 Ablation of the Ventral Root and Response of the Primary Sympathetic Ganglia

Supplementary Movie 8 Reposition of the Ventral Root and Response of the Primary Sympathetic Ganglia

Supplementary Movie 9 Ectopic Transplantation of BDNF bead and Response of the Primary Sympathetic Ganglia

Supplementary Movie 10 Blocking of BDNF Signaling and Response of the Primary Sympathetic Ganglia
