# Supplementary material for: TrkB/BDNF signalling patterns the sympathetic nervous system
Source: Nat Commun. 2015 Sep 25;6:8281. doi: 10.1038/ncomms9281 (PMC4586040; doi:10.1038/ncomms9281)
Supplement: Supplementary Information — Supplementary Figure 1 [file ncomms9281-s1.pdf]

a

Cell type	Stage (HH)	Developmental timing	# of embryos/ biol. replicates	Cells per replicate	Total cells
SG	24,26	Prior and mid-dorsal migration	10	10	10
Ventral neural tube	25	Mid-dorsal migration	10	10	10
Ventral root	25	Mid-dorsal migration	10	10	10

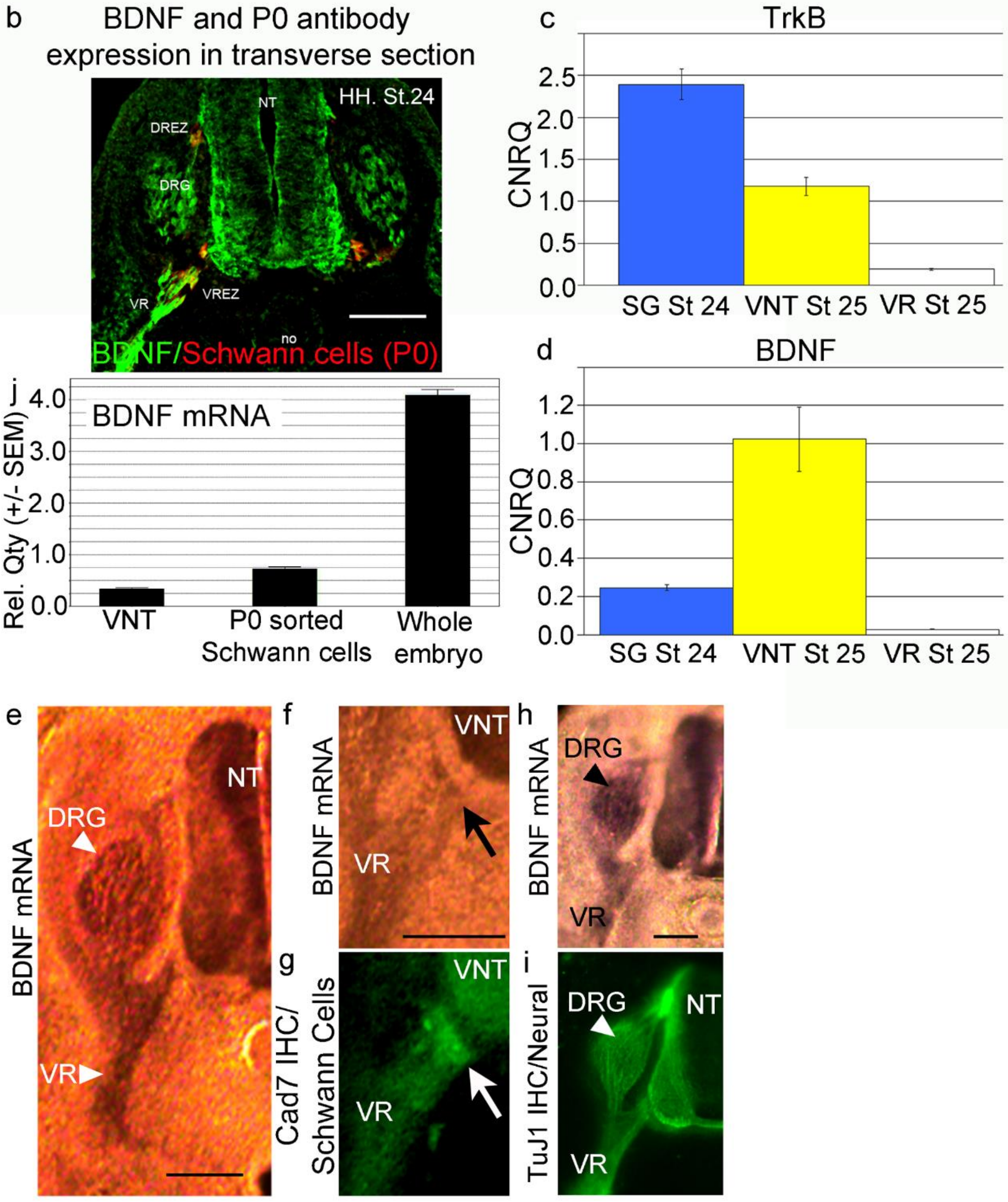

Supplementary Figure 1. **LCM profiling information and BDNF expression expression analysis.** (a) LCM profiling information. (b) BDNF and P0 immunohistochemistry at HH. St.24 in transverse section. BDNF (green) is expressed in the ventral neural tube, DRG and along the ventral root. P0 (red, Schwann cells) is expressed at the DREZ and the VREZ. NT=neural tube, no=notochord, DRG=dorsal root ganglia, VR=ventral root, DREZ=dorsal root entry zone, VREZ=ventral root entry zone. (c-d) qPCR results of TrkB and BDNF expression from LCM isolated cells. (e) in situ hybridization for BDNF mRNA at E4 shows expression in the DRG, NT and VR. (f-g) BDNF ISH followed by Cad7 antibody (Schwann cells) staining shows overlap between BNDF mRNA and Cad7 protein at the ventral root exit zone (black and white arrows). (h-i) BDNF ISH followed by Tuj1 antibody staining (neural) shows overlap between BDNF mRNA and Tuj1 protein in the DRG, NT, ventral NT and VR. (j) qPCR analysis on E4 FACS sorted cells, shows BDNF mRNA expression in ventral neural tube cells, P0 sorted Schwann cells, and whole embryo. Scale Bars=100 um.
